# Fluorescence correlation spectroscopy reveals a cooperative unfolding of monomeric amyloid-β 42 with a low Gibbs free energy

**DOI:** 10.1038/s41598-017-02410-y

**Published:** 2017-05-19

**Authors:** Mario Schneider, Stefan Walta, Chris Cadek, Walter Richtering, Dieter Willbold

**Affiliations:** 1 Institut für Physikalische Biologie, Heinrich-Heine-University Düsseldorf, Düsseldorf, Germany; 2 Institute of Physical Chemistry, RWTH Aachen University, JARA - Soft Matter Science, Aachen, Germany; 3 Institute of Complex Systems, Structural Biochemistry (ICS-6), Research Center Jülich, Jülich, Germany

## Abstract

The amyloid-beta peptide (Aβ) plays a major role in the progression of Alzheimer’s disease. Due to its high toxicity, the 42 amino acid long isoform Aβ42 has become of considerable interest. The Aβ42 monomer is prone to aggregation down to the nanomolar range which makes conventional structural methods such as NMR or X-ray crystallography infeasible. Conformational information, however, will be helpful to understand the different aggregation pathways reported in the literature and will allow to identify potential conditions that favour aggregation-incompetent conformations. In this study, we applied fluorescence correlation spectroscopy (FCS) to investigate the unfolding of Alexa Fluor 488 labelled monomeric Aβ42 using guanidine hydrochloride as a denaturant. We show that our Aβ42 pre-treatment and the low-nanomolar concentrations, typically used for FCS measurements, strongly favour the presence of monomers. Our results reveal that there is an unfolding/folding behaviour of monomeric Aβ42. The existence of a cooperative unfolding curve suggests the presence of structural elements with a Gibbs free energy of unfolding of about 2.8 kcal/mol.

## Introduction

Aggregation of amyloid-beta (Aβ) is believed to be one of the key processes in the development and progression of Alzheimer’s disease (AD). Monomeric Aβ is formed by cleavage of the amyloid precursor protein (APP) by β- and γ-secretases. Depending on the exact cleavage sites of the secretases, different isoforms of the monomeric Aβ evolve and are released into the extracellular space. The high aggregation propensity of Aβ42 even at low micromolar concentrations is a major problem to elucidate the monomer structure in purely aqueous solutions by conventional techniques such as nuclear magnetic resonance (NMR) spectroscopy or X-ray diffraction. Therefore, information about Aβ42 monomer conformation exists so far only from solutions containing hexafluoroisopropanol (HFIP)^1, 2^. However, these conditions are far from being physiological, especially because typical Aβ42 concentrations *in vivo* are in the nanomolar range^3^. Thus, there is not much experimental evidence on the structural properties of Aβ monomers. Understanding the structural conversions of Aβ42 during aggregation can be the basis for drug development and AD treatment. It is suggested that the Aβ42 monomer might exist in equilibrium between a folded and unfolded conformer^4^, but to date there is no substantial experimental evidence for this hypothesis.

In this study, we applied fluorescence correlation spectroscopy (FCS) to follow changes in the hydrodynamic radius of dye-labelled Aβ42 and thus to be able to observe its unfolding transition with increasing guanidine hydrochloride (GndHCl) concentrations. Since FCS measurements are typically performed at low nanomolar concentrations, this technique is well-suited to study dye-labelled Aβ42 monomers.

Our findings demonstrate that Aβ42 shows a cooperative unfolding pattern when denatured with GdnHCl under near-physiological conditions. The Gibbs free energy of unfolding was determined to be about 2.8 kcal/mol, thereby indicating that stable structural elements are present.

## Materials and Methods

### C(0)Aβ42 dye labelling and purification

We dissolved lyophilized recombinant Aβ42 with an additional cysteine residue at the N-terminal end (position zero) “C(0)Aβ42” (preparation is described in the Supplementary Information), in 6 M GdnHCl (pH 7.4) containing a 10-fold molar excess of tris(2-carboxyethyl)phosphin (TCEP). 1 mg of Alexa Fluor 488 maleimide (Molecular Probes, Eugene, OR, USA) was dissolved in 100 µL DMF (*N,N*-Dimethylformamide Uvasol for spectroscopy, Merck, Darmstadt, Germany) and added to the reaction mixture in order to maintain a 5-fold molar excess of the dye. The labelling reaction at the N-terminal cysteine residue was performed at room temperature overnight. Hereafter, unreacted dye was separated from labelled C(0)Aβ42 (termed AF488-C(0)Aβ42) by reversed-phase high performance liquid chromatography (rp-HPLC) with 29% Acetonitrile/H_2_O + 0.1% TFA and at 80 °C. Furthermore, the resulting dye-conjugate fraction was lyophilized (SpeedVac (AVC 2-18) with cold-trap LT-105, Christ, Osterode am Harz, Germany) and stored at −80 °C until further use.

Our dye-conjugate variant of Aβ42 with an additional cysteine at the N-terminus is expected to have the same structural properties as the unmodified Aβ42. The insertion of the uncharged amino acid cysteine at the N-terminus, which is known to be unstructured^1, 5^, is not expected to change the properties of our monomer. Compared with the dye molecule, the additional cysteine contributes less molecular weight that the dye itself and acts as a linker between the dye and the Aβ42 to prevent interference between the dye and the Aβ peptide^6^. Based on the most direct and important property of Aβ42 we did not find any drastic change. In particular, we found that the dye-conjugate of C(0)Aβ42 is able to aggregate exactly as Aβ42, as shown in the size exclusion chromatography (SEC) (Fig. S1) of C(0)Aβ42 indicating aggregates thereof eluting earlier than the C(0)Aβ42 monomers.

### Preparation of guanidine hydrochloride solutions

All solutions were prepared with Milli-Q water (Merck Millipore, Darmstadt, Germany) and high-purity 8 M GdnHCl stock solution. The 8 M GdnHCl stock solution was used to prepare a 6 M GdnHCl solution in 125 mM phosphate buffer at pH 7.4. The resulting solution was then further used to prepare GdnHCl solutions ranging from 0 to 6 M in 125 mM phosphate buffer (pH 7.4) for the unfolding experiments. The GdnHCl concentration of each solution was checked by refractive-index measurements (Refractometer, Bausch & Lomb, Rochester, NY, USA) using an empirical relation between GdnHCl concentration and refractive index, as described by Nozaki *et al*.^7^. Additionally, we measured the kinematic viscosity (micro-Ostwald Viscosimeter with capillary type I, LAUDA Dr. R. Wobser GmbH & Co. KG, Lauda-Königshofen, Germany) and the density (Density meter DMA 5000, Anton Paar, Scharnhausen, Germany) of each of these GdnHCl solutions to obtain the dynamic viscosity at 23 °C. This was needed in order to convert the diffusion coefficient into a hydrodynamic radius, as shown in the Supplementary Information.

### 1-focus fluorescence correlation spectroscopy measurements

1-focus fluorescence correlation spectroscopy (1fFCS) measurements were performed on a home-built confocal fluorescence detection setup equipped with a pulsed laser diode (λ = 470 nm, LDH-P-C-470, PicoQuant, Berlin, Germany). The laser light is forwarded into the microscope via a dichroic mirror (dichroic mirror z470/635, AHF Analysentechnik, Tübingen, Germany). A water-immersion objective (UplanApo 60 × 1.2 W, Olympus, Melville, NY, USA) focuses the laser beam into the sample solution where fluorescent molecules are excited. Fluorescence light is collected by the same objective in reverse direction, passing through the dichroic mirror and focused onto a pinhole of 100 µm diameter by a tube lens. A polarising beam splitter separates photons according to their polarisation. Each beam is subsequently focused onto a single-photon avalanche detector (SPAD) detector (PDM, MPD, Bolzano, Italy). For fluorescence correlation spectroscopy (FCS) analysis, the signals from both detectors were cross-correlated to remove detector artifacts such as afterpulsing^8^.

FCS measurements were conducted in a 389 well-plate (384 Well Greiner Microplate, Greiner Bio-One, Frickenhausen, Germany) covered with foil to prevent solvent evaporation. The correction collar was adjusted to gain maximum intensity when measuring a concentrated solution (~10 nM) of Alexa Fluor 488 (AF488). A laser power of about 20 µW was chosen to prevent triplet formation and saturation effects. The concentration of AF488-C(0)Aβ42 was about 3 nmol/L. The correlation and the following fitting were performed using custom software written in MATLAB (The MathWorks, Natick, MA, USA). A typical FCS measurement contained more than 20 million detection events. This data was subsequently split into subpackages of 2 million events resulting in 10 correlation curves for each GdnHCl concentration. The correlation curves were fitted by a one-component diffusion model and subsequently converted into hydrodynamic radii by the Stokes-Einstein relation. Further details on data fitting and evaluation can be found in the Supplementary Information.

### Dual-focus fluorescence correlation spectroscopy measurements

Dual-focus fluorescence correlation spectroscopy (2fFCS) measurements were performed on a time-resolved confocal fluorescence microscopy system (MicroTime 200 with dual-focus option, PicoQuant, Berlin, Germany). For excitation, two identical laser diode heads (λ = 470 nm, LDH-P-C-470, PicoQuant, Berlin, Germany) provide synchronised laser pulses with alternating orthogonal polarisation. According to this polarisation, the laser pulses are deflected in two slightly different directions by a Nomarski prism (U-DICTHC, Olympus, Melville, NY, USA), which is placed in front of the water-immersion objective (UPLSAPO 60xW, 1.2 N.A., Olympus, Melville, NY, USA). In this way, two overlapping but laterally shifted foci are generated in the sample. Because the lateral distance remains constant over time for a given wavelength, absolute values of the diffusion coefficient can be measured^9^.

2fFCS measurements were conducted in sealed sample cells at 23 °C, whereas an absolute accuracy of 0.1 °C is achieved by homemade temperature regulation^10^. The total laser power was adjusted to approximately 50 µW, before the laser light is reflected towards the objective. The measurement time was adjusted to detect 4 × 10^6^ photons per point at least. The detected photons were divided into 2 × 10^6^ photon subpackages. Several points were recorded for each sample. The resulting autocorrelation and cross-correlation functions were fitted by a one-component diffusion model. The obtained diffusion coefficients were averaged over all measured data points and converted into hydrodynamic radii by the Stokes-Einstein relation. Further details on the 2fFCS setup, data fitting and evaluation can be found in the Supplementary Information.

### Photon Counting Histogram analysis

Photon counting histogram (PCH) analysis was performed to check if the amyloid beta molecule solely exists in its monomeric form under low-nanomolar concentrations. PCH analysis is a powerful tool to discriminate different molecules based on their brightness. Müller *et al*. demonstrated that PCH is capable of distinguishing singly and doubly labelled proteins^11^. Thus, PCH is complementary to FCS which is not able to resolve two fluorescent molecules of similar size^12^. Additionally, PCH is especially useful when molecules tend to form oligomers^13^. The basics of PCH analysis are briefly described in the Supplementary Information. For more details, we refer to the paper by Chen *et al*.^14^.

### Unfolding curve data analysis

By plotting the hydrodynamic radius *R*_H_ versus the denaturant concentration, an experimental curve was obtained which is referred to as experimental unfolding curve subsequently. A two-state unfolding model was applied to fit the experimental unfolding curves (equation (1)) by using a Levenberg-Marquardt simplex algorithm in Origin (OriginLab, Northampton, MA, USA).

1 $${R}_{{\rm{H}}}=\frac{{R}_{H,N}+{R}_{H,U}\exp (-\frac{{\rm{\Delta }}{G}^{{H}_{2}O}-m\,[D]}{RT})}{1+\exp (-\frac{{\rm{\Delta }}{G}^{{H}_{2}O}-m\,[D]}{RT})}$$

*R*_H,N_ and *R*_H,U_ denote the hydrodynamic radius of the native and unfolded form of the peptide, respectively, *T* is absolute temperature in Kelvin and *R* is universal gas constant. ${\rm{\Delta }}{G}^{{H}_{2}O}$ denotes the Gibbs free energy in the absence of denaturant and can be used to assess the structural stability of a peptide^15^. *m* is a measure of cooperativity of the unfolding transition and is proportional to the change in the solvent-accessible surface area (ΔSASA) when going from the native to the denatured conformation^16^. This will be discussed in more detail in the Supplementary Information. The denaturant concentration is given by [D]. Equation (1) incorporates the linear extrapolation model which assumes a linear relationship between the Gibbs free energy Δ*G* and the denaturant concentration^17^:

2 $${\rm{\Delta }}G={\rm{\Delta }}{G}^{{H}_{2}O}-m\,[D]$$

For a better comparability between several independent measurement series, the hydrodynamic radii from the FCS unfolding experiments were also transformed into the fraction of denatured peptide *f*_exp_ by the following equation:

3 $${f}_{\exp }=\frac{{R}_{{\rm{H}}}-{R}_{{\rm{H}},{\rm{\min }}}}{{R}_{{\rm{H}},{\rm{\max }}}-{R}_{{\rm{H}},{\rm{\min }}}}$$

*R*_H,min_ and *R*_H,max_ denote the smallest and largest measured hydrodynamic radius of the peptide, respectively. With the fractions of the native and unfolded form of the peptide, *f*_N_ and *f*_U_, equation (1) turns to:

4 $$f=\frac{{f}_{N}+{f}_{U}\exp (-\frac{{\rm{\Delta }}{G}^{{H}_{2}O}-m\,[D]}{RT})}{1+\exp (-\frac{{\rm{\Delta }}{G}^{{H}_{2}O}-m\,[D]}{RT})}$$

## Results and Discussion

### AF488-C(0)Aβ42 is monomeric under FCS measuring conditions

**Figure 1:**
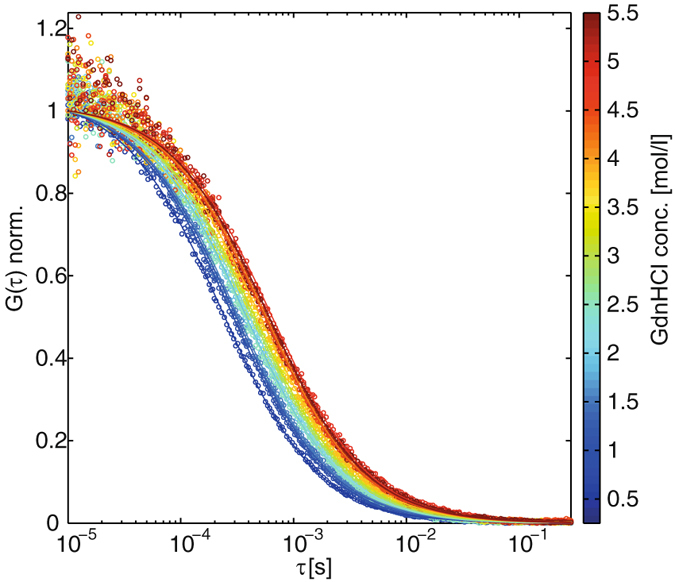
Normalized correlation curves (○) with their corresponding best-fit lines (−) of AF488-C(0)Aβ42 under denaturing conditions of increasing GdnHCl concentrations and based on a one-component diffusion model (equation (S1) in the Supplementary Information). The shift towards larger correlation times can mainly be attributed to (i) the increasing viscosity of the GdnHCl solutions and (ii) an increase of the hydrodynamic radius due to the unfolding process.

Purely monomeric AF488-C(0)Aβ42 was prepared from recombinant C(0)Aβ42 after AF488 coupling to the cysteine residue and subsequent size exclusion chromatography (SEC) (Fig. S1 in the Supplementary Information). The time between SEC purification and the FCS unfolding measurements ranged from minutes to hours. We have not observed any dependence of the experimental results on the lag time between SEC purification and experiment start. There was no indication for the presence of AF488-C(0)Aβ42 aggregates from FCS analysis (Figs 1 and S2).

**Figure 2:**
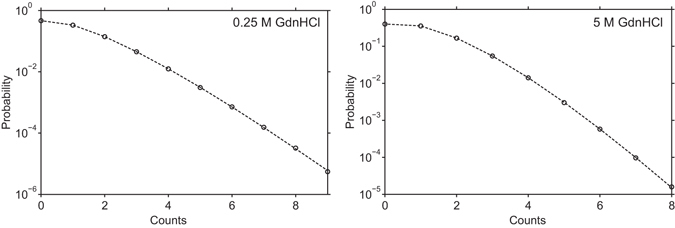
Photon counting histograms of AF488-C(0)Aβ42 in 0.25 M GdnHCl solution (left) and in 5 M GdnHCl solution (right). 0.25 M GdnHCl was the lowest GdnHCl concentration applied in the 1fFCS measurements whose data was used to generate the photon counting histograms. Each dashed line in this figure represents a fit to a one-component model. A reduced chi-squared of ${{{\rm{\chi }}}_{{\rm{r}}}}^{2}$ = 0.6 and 1.2, respectively, indicates an excellent fit to this model where the F-value (equation (S16)) was determined from a separate measurement with free AF488 dye.

Hence, in order to be sure that the unfolding transition is solely due to conformational changes of the monomers, we performed PCH analysis on the data set of the FCS unfolding experiments, particularly at the lowest GdnHCl concentration (0.25 M) and at a high GdnHCl concentration (5 M). In case there would have been oligomers present in the solution, a one-component fit to the experimental PCH would have failed and resulted in a reduced chi-squared much higher than one. Figure 2 reveals that fitting the experimental PCH of AF488-C(0)Aβ42 with a one-component model yielded a good fit with a reduced chi-squared close to one over the entire GdnHCl concentration range.

In addition, the brightness values of free AF488 dye and AF488-C(0)Aβ42 were almost identical at 0.25 M and 5 M GdnHCl, respectively (Table 1). A substantial amount of oligomeric species would have yielded a higher brightness of AF488-C(0)Aβ42 compared to the free dye^13^. The 2.5–3-fold smaller brightness at 5 M GdnHCl results from a bathochromic shift in the excitation and emission spectra (Fig. S8), which leads to a decrease in the detected intensity since the excitation and emission filter set was chosen according to the non-shifted spectrum at zero molar GdnHCl. It shall be noted that the verification of the monomeric form was performed on the same data set, which was finally used to construct the unfolding curve. This is in contrast to other methods such as circular dichroism (CD) spectroscopy or 4,4′-bis-1-anilinonaphthalene-8-sulfonate (Bis-ANS) fluorescence in which the verification is done on a separate instrument (*e.g*. SEC) after the actual unfolding measurements (see *e.g*. ref. 18).

**Table 1:** Comparison of the brightnesses of free AF488 dye and AF488-C(0)Aβ42 in 0.25 M and 5 M GdnHCl solution obtained from PCH analysis (Fig. 2).

sample	AF488		AF488-C(0)Aβ42	
GdnHCl conc. [M]	0.25	5	0.25	5
brightness [kHz]	16.23 (±0.22)	6.36 (±0.43)	18.73 (±2.23)	6.32 (±0.18)

### Monomeric AF488-C(0)Aβ42 shows a cooperative unfolding transition

**Figure 3:**
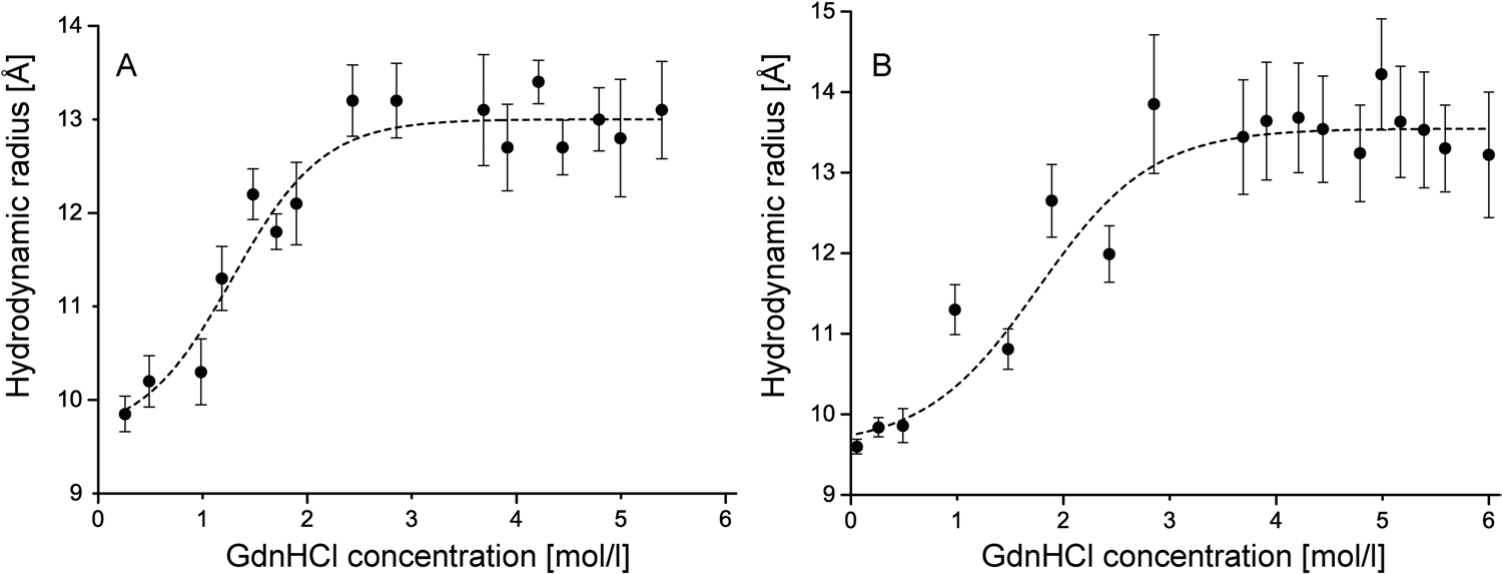
AF488-C(0)Aβ42 unfolding curves as obtained by the analysis of a single 1fFCS measurement series (**A**) and as obtained by the analysis of a single 2fFCS measurement series (**B**). The dashed lines are best-fit lines to the two-state unfolding model (equation (1)). The hydrodynamic radius is plotted as a function of the GdnHCl concentration. The error bars of the unfolding curves were derived as the standard deviation of the fitting results of 10 correlation curves per data point.

Upon unfolding in GdnHCl, the increasing hydrodynamic radii, as well as the increasing viscosity of the solutions, leads to a right-shift of the correlation curves, as shown in Fig. 1. For quantifying the unfolding effect, the correlation curves were fitted by a one-component diffusion model (equation (S1)) and the corresponding diffusion coefficients were converted into hydrodynamic radii via the Stokes-Einstein relation. Plotting the hydrodynamic radii as a function of the GdnHCl concentration yields the appropriate unfolding curves shown in Fig. 3A and B.

We measured a whole unfolding series of AF488-C(0)Aβ42 using 2fFCS and compared the corresponding unfolding curve with one obtained by 1fFCS in order to validate that the 1fFCS results are not biased by refractive-index changes. Both unfolding curves show a similar course with a transition region between 1 and 2.5 M GdnHCl. The hydrodynamic radius *R*_*H,N*_ at about zero molar GdnHCl corresponds to the hydrodynamic radius of the native conformation. The *R*_*H,N*_- value is about 9.6 Å for both methods, which is in good agreement with the results reported in studies that characterized Aβ isoforms^19–21^. The hydrodynamic radius of the unfolded conformation *R*_H,U_ can be extracted from the unfolding curve at high GdnHCl concentrations (3 to 6M) and is about 13.0 Å (A) or 13.6 Å (B). Because Aβ42 is small and believed to be unstructured to some extent the absolute change in hydrodynamic radius upon unfolding was expected to be much smaller than for other proteins^22–24^. Our results confirm our assumption.

The steepness of the unfolding curve in the transition region contains information about the structural stability of the peptide. This information is enclosed in the *m*-value and ${\rm{\Delta }}{G}^{{H}_{2}O}$, respectively (equations (1) and (2)), whereas the latter one reports on the stability of the native conformation of a protein in the absence of denaturant^15^ and can thus, for example, be used to compare protein mutants with their corresponding wild-type^25^. Both parameters can be extracted by fitting the experimental unfolding curve to a two-state unfolding model (equation (1)). As presented in Table 2, the parameters ${\rm{\Delta }}{G}^{{H}_{2}O}$ and *m* are very close together.

**Table 2:** Fit parameter from fitting the unfolding curves (Fig. 3A and B) to the two-state unfolding model (equation (1)).

method	${\boldsymbol{\Delta }}{{\boldsymbol{G}}}^{{{\boldsymbol{H}}}_{{\bf{2}}}{\boldsymbol{O}}}$ [kcal/mol]	*m* [kcal/mol/M]
1fFCS	2.6 (±0.7)	1.7 (±0.5)
2fFCS	1.9 (±0.5)	1.1 (±0.3)
average	2.3 (±0.6)	1.4 (±0.4)

It shall be mentioned that the 1fFCS and 2fFCS unfolding curves in Fig. 3A and B are based on a single measurement series each, which results in small error bars of the unfolding curves and correspondingly in small errors of the fit parameters. In this case, the error bars of the unfolding curves were derived as the standard deviation of the fitting results of 10 correlation curves per data point. Within one measurement series, the error bars tend to increase with increasing GdnHCl concentrations. The reasons for this may be manifold and are discussed in the Supplementary Information (Figs S3–S5 and S8). Furthermore, it was checked whether the change in hydrodynamic radius is associated with the unfolding of the Aβ42 peptide and not to structural changes of the conjugated AF488 dye. For this purpose, the free AF488 dye was measured at several GdnHCl concentrations up to 6 M and no increase in hydrodynamic radius was observed (Fig. S6).

**Figure 4:**
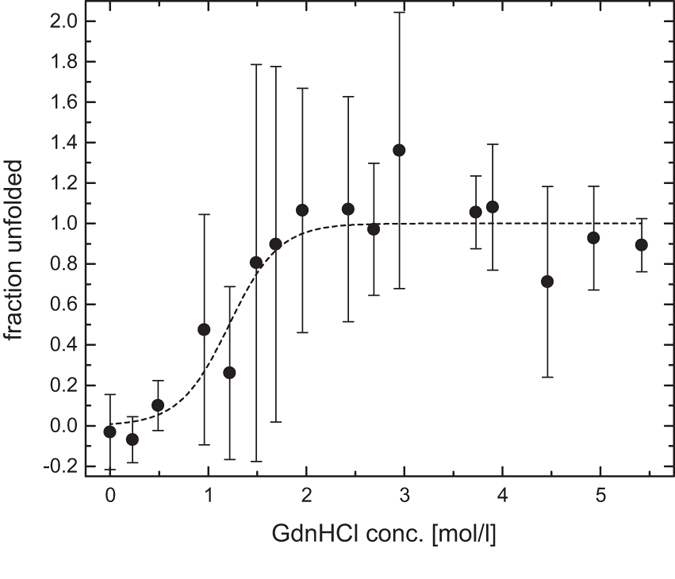
Unfolding curve of AF488-C(0)Aβ42 from three independent 1fFCS and one 2fFCS measurement series with their corresponding best-fit line to the two-state unfolding model (equation (4)). Each data point is the average of at least three hydrodynamic radii, which were transformed into the fraction of denatured peptide according to equation (3). The error bars were calculated as the standard deviation between these independent measurements.

The experimental data was normalized by using equation (3). Consequently, the fraction of unfolded peptide is plotted as a function of the GdnHCl concentration, leading to the unfolding curve presented in Fig. 4. Figure 4 shows the unfolding curve of AF488-C(0)Aβ42 from three independent 1fFCS and one 2fFCS measurement series. The hydrodynamic radii of the folded and the unfolded states are fully reproducible at around 9.6 and 13 Å, respectively, but the hydrodynamic radii around the transition points showed larger variations between independent measurement series. Thus, although each single unfolding curve is within the error margins of the other unfolding curves, the combined unfolding curve results in error bars that are larger around the transition point than at low and high GdnHCl concentrations. Therefore, the error bars of the corresponding fit parameters in Table 3, which are in line with the values calculated in Table 2, are also larger. AF488-C(0)Aβ42 is predominantly in its native conformation up to 0.5 M GdnHCl. A relatively steep transition region between 1 and 2 M GdnHCl is followed by a plateau above 2 M at which the AF488-C(0)Aβ42 was completely unfolded. Each data point was measured at least in triplicate. Thus, the corresponding error bars in Fig. 4 were calculated as the standard deviation between these independent measurements.

**Table 3:** Fit parameters from fitting the unfolding curve (Fig. 4) to the linear extrapolation model (equation (3)). The fit was performed on the averaged unfolding curve.

${\boldsymbol{\Delta }}{{\boldsymbol{G}}}^{{{\boldsymbol{H}}}_{{\bf{2}}}{\boldsymbol{O}}}$ [kcal/mol]	*m* [kcal/mol/M]
2.8 (±1.2)	2.3 (±0.9)

The errors denote the standard errors of the fit parameters.

The ${\rm{\Delta }}{G}^{{H}_{2}O}$-value calculated in Table 3 is reasonably low and indicates that, from a thermodynamic point of view, there is energetically no considerable difference between the unfolded conformation and the native conformation (free in solution). Depending on the free energy of activation for the interconversion of both conformations, the equilibrium will quickly be achieved once the AF488-C(0)Aβ42 is formed in its native conformation. Buell *et al*. showed that the Gibbs free energy of activation for the conformational change of native Aβ42 to a beta-sheet conformation (as found in fibrils) is very low (1.4 kcal/mol)^26^. If we assume a similar value for the interconversion between the native and the unfolded state, chemical equilibrium will be reached quickly. In addition, it was shown that a low ${\rm{\Delta }}{G}^{{H}_{2}O}$-value corresponds to a high aggregation propensity^27^, which goes well with the known properties of Aβ42^28^.

It is worth mentioning that our ${\rm{\Delta }}{G}^{{H}_{2}O}$-value is larger than typical values for the strength of hydrogen bonds within proteins (≈0.5–1.5 kcal/mol^29^). Hence, the native conformation might contain several hydrogen bonds which leads to some residual structure, *e.g*. a short beta-sheet at the C-terminus^28^ or a molten globule in which the overall structure is denatured with some residual structural elements^30^. In a molten globule many hydrophobic residues can be surface-exposed and lead to a sticky behaviour of the corresponding peptide, which also goes well with the properties of Aβ42^31, 32^. In any case, the results do not agree with a completely randomly coiled peptide as opposed to what is partially claimed in the literature^33, 34^.

Our results show a cooperative unfolding of monomeric AF488-C(0)Aβ42 with a Gibbs free energy of about 2.8 kcal/mol. The peptide contains some residual structural elements and is thus not completely random coiled. We think that monomeric Aβ42 adopts a molten globule conformation under physiological conditions. A future study is going to examine this issue, which is, however, beyond the scope of this paper.

## Conclusions

With this study, we explored the unfolding of dye-labelled monomeric amyloid-β 42 (Aβ42) peptide under near-physiological conditions using fluorescence correlation spectroscopy. We showed that this technique is able to track the tiny changes of the hydrodynamic radius when monomeric Aβ42 unfolds in guanidine hydrochloride. We found that Aβ42 shows a cooperative unfolding, which clearly suggests the presence of structural elements. However, the small value of the Gibbs free energy of unfolding (≈2.8 kcal/mol) indicates that only a low amount of residual structure is present and that Aβ42 is mainly unfolded. Our results hint that monomeric Aβ42 adopts a molten globule conformation.

## Electronic supplementary material

Supporting Information
