# Supplementary material for: Fluorescence correlation spectroscopy reveals a cooperative unfolding of monomeric amyloid-β 42 with a low Gibbs free energy
Source: Sci Rep. 2017 May 19;7:2154. doi: 10.1038/s41598-017-02410-y (PMC5438374; doi:10.1038/s41598-017-02410-y)
Supplement: Supplementary file 1 — Supporting Information [file 41598_2017_2410_MOESM1_ESM.pdf]

# Fluorescence correlation spectroscopy reveals a cooperative unfolding of monomeric amyloid-β 42 with a low Gibbs free energy

Mario Schneider, Stefan Walta, Chris Cadek, Walter Richtering, Dieter Willbold

## Supplementary Information

### Peptide expression and purification

A variant of Aβ42 with an additional cysteine at the N-terminus (C(0)Aβ42), was expressed in *E. coli* Bl21-DE3 as a fusion protein[1]. The pRSET vector containing the fusion construct was kindly provided by Rudolf Glockshuber. The cysteine codon was introduced in front of the alanine codon of the Aβ42-sequence via QuikChange-PCR (QuikChange Lightning Site-Directed Mutagenesis Kit, Agilent Technologies, Santa Clara, CA, USA). The subsequent recombinant expression and purification of the fusion protein, as well as the tobacco etch virus (TEV) cleavage, were similarly performed to the procedure described in Finder *et al.*[1].

Briefly, *E.coli* cells were transformed with the pRSET plasmid, and a single colony was subsequently chosen to inoculate a 5 mL starter culture in Luria broth medium containing 100 µg/mL Ampicillin (Sigma-Aldrich, Steinheim, Germany). This culture was incubated at 37 °C overnight. 1 L of culture medium was inoculated with the starter culture to an optical density of $OD_{600nm}$ = 0.1 and cultivated at 37 °C to an optical sensity of $OD_{600nm}$ = 3. Afterwards, protein expression was induced with 1 mM Isopropyl-ß-D-thiogalactopyranosid (IPTG, Thermo Fisher Scientific, Waltham, MA, USA), and cells were further cultivated for 16 h at 30 °C. Cells were harvested by centrifugation at 4000 g and 4 °C. The cell pellet was resuspended in 4 mL ice-cold lysis buffer (30 mM sodium phosphate buffer, 300 mM NaCl, pH 8, 2.4 U/mL Benzonase Nuclease, 2 mM $Mg^{2+}$, 1 cOmplete Protease Inhibitor Cocktail Tablet - EDTA free) per mg cell pellet and lysed by a cell disruptor at 2.9 kbar. After addition of GdnHCl (final concentration 6 M) the solution was incubated for 90 min at room temperature. This solution was then centrifuged at 197807 g and 4 °C for 45 min, and the supernatant was subsequently purified by nickel–nitrilotriacetic acid (Ni-NTA) chromatography. The chromatography system (Äkta Prime with column XK16/20, GE Healthcare, Munich, Germany) was equilibrated with lysis buffer containing 6 M GdnHCl. Then, the sample was loaded onto the column followed by a washing step with lysis buffer. The fusion protein was eluted by a rising gradient of elution buffer (30 mM NaPi, 300 mM NaCl, 500 mM Imidazole, 6 M GdnHCl) at 2 mL/min flow rate.

Later, the fusion protein was purified by rp-HPLC (HPLC 1260 Infinity with Zorbax-SB300 C8-column, Agilent, Böblingen, Germany). Therefore, the column was tempered to 80 °C and equilibrated with 29 % (vol/vol) Acetonitrile (VWR, Darmstadt, Germany), 0.1 %(vol/vol) TFA (Trifluoroacetic acid ReagentPlus, Sigma Aldrich, St. Louis, USA). Sample elution was performed under isocratic conditions at 29 % (vol/vol) Acetonitrile, 0.1 %(vol/vol) TFA at 4 mL/min flow rate. The fusion protein in the Acetonitrile-water-mixture was lyophilized and subsequently cleaved by TEV-protease in digestion buffer (100 mM Tris-HCl pH 7.8, 50 mM Tris(2-carboxyethyl)phosphine (TCEP, hydrochloride BioUltra, Sigma-Aldrich)) containing 0.05 mol equivalent TEV-Protease at 4 °C overnight. To separate the C(0)Aβ42 from the residual protein tag another rp-HPLC step was performed as described above. The resulting fractions were lyophilized and stored at -80 °C until further use. We confirmed the molecular weight by matrix-assisted laser desorption/ionisation time-of-flight mass spectrometry (MALDI-TOF MS).

Another (independent) sample of C(0)Aβ42 was obtained as a gift from Isoloid GmbH (Düsseldorf, Germany). The peptide labelling was performed as described in the main text.

**AF488-C(0)Aβ42 monomerisation**

AF488-C(0)Aβ42 was dissolved in a 6 M GdnHCl solution and subsequently applied to a SEC system (Agilent 1100 HPLC with Superdex 75 3.2/300 column GE Healthcare, Munich, Germany). Elution was performed with 125 mM phosphate buffer (pH 7.4) at a flow rate of 0.1 mL/min. Due to the high amount of GdnHCl the amounts of oligomers is remarkably low (Fig. S1).

The AF488-C(0)Aβ42 monomer yield was typically about 5 µM. The monomer fraction was collected in Protein LoBind tubes (Eppendorf AG, Hamburg, Germany). The following addition of 8 M GdnHCl (8 M Guanidine-HCl solution, Thermo Scientific, Waltham, MA, USA) to a final concentration of 6 M GdnHCl was supposed to stabilise the monomers and prevent aggregation after fractionation. The resulting solution, with a final monomer concentration of about 1 µM or less, was the stock for fluorescence fluctuation measurements and could be stored at -20 °C without actually freezing.

**1-focus fluorescence correlation spectroscopy measurements**

Each correlation curve was fitted to a single species diffusion model:

$$G(\tau) = \frac{1}{N_{\mathrm{eff}}}\left[1+\frac{\tau}{\tau_{\mathrm{D}}}\right]^{-1}\left[1+\frac{\tau}{s^2\tau_{\mathrm{D}}}\right]^{-1/2} \qquad \text{(S1)}$$

Herein $\tau_D$ denotes the diffusion time, $N_{eff}$ the number of molecules in the effective volume and *s* the eccentricity of the effective volume which is approximated by a three-dimensional Gaussian:

$$\mathrm{PSF}(r) = \exp\left(\frac{-2\left(x^2+y^2\right)}{\omega_{xy}^2}\right)\exp\left(\frac{-2z^2}{z_0^2}\right) \quad \text{(S2)}$$

*s* can be expressed in terms of the lateral $\omega_{xy}$ and axial radius $z_0$ of the effective volume:

$$s = \frac{z_0}{\omega_{xy}} \quad \text{(S3)}$$

*s* was determined for each GdnHCl concentration by a calibration measurement with AF488 maleimide whose diffusion coefficient (*D* = 275 ± 4 µm²/s) in water at 25 °C was determined by 2fFCS. In order to obtain the diffusion coefficient of AF488 at a given GdnHCl concentration *X* and temperature *T* we used the following relation:

$$D_{X,T} = D_{ref}\frac{T}{T_{ref}}\frac{\eta_{ref}}{\eta_X} \quad \text{(S4)}$$

Herein $D_{ref}$ denotes the diffusion coefficient of AF488 in water at $T_{ref} = 298.15K$ and the corresponding dynamic viscosity $\eta_{ref}$.

*s* was fixed for the analysis of the correlation curves from the AF488-C(0)Aβ42 measurements, whereas $\tau_D$ and $N_{eff}$ were left as free parameters. We converted the diffusion time $\tau_D$ into a diffusion coefficient *D* *via* the following relation:

$$D = \frac{\omega_{xy}^2}{4\tau_D} \quad \text{(S5)}$$

*D* was finally converted into a hydrodynamic radius $R_H$ with the Stokes-Einstein relation:

$$R_H = \frac{k_B T}{6\pi\eta D} \quad \text{(S6)}$$

**Dual-focus fluorescence correlation spectroscopy measurements**

The fluorescence light is collected by the same objective and focused onto a confocal pinhole of 75 µm diameter. After passing through an emission filter (HQ520/40m, AHF-Analysentechnik, Tübingen, Germany), the light is guided onto two SPAD detectors (PDM

series, Micro Photon Devices, Bolzano, Italy) by a non-polarising beam splitter cube. Every detected photon is referred to its arrival time with respect to the start of the experiment and the last laser pulse by a dedicated time-correlated single photon counting (TCSPC)[2] electronics (PicoHarp 300, PicoQuant, Berlin, Germany). That way, it is known in which focus the fluorescence photon was generated. Thus, autocorrelation functions (ACFs) of the two foci and cross-correlation functions (CCFs) between them are calculated with an algorithm based on a multiple-tau approach[3]. Afterpulsing artifacts are avoided by correlating photons from different detectors[4].

For subsequent data fitting, the shape of the molecular detection function (MDF) in both foci has to be taken into account. The MDF gives the probability to detect a photon at a certain position in one of the detection volumes and can be described by a two-parameter model based on a modified three-dimensional elliptic Gaussian[5]:

$$U(\vec{r}) = \frac{\kappa(z)}{w^2(z)} \exp\left[\frac{-2}{w^2(z)}\left(x^2 + y^2\right)\right] \qquad \text{(S7)}$$

with the beam waist function:

$$w(z) = w_0\left[1 + \left(\frac{\lambda_{\mathrm{ex}} z}{\pi w_0^2 n}\right)^2\right]^{1/2} \qquad \text{(S8)}$$

and amplitude function:

$$\kappa(z) = 1 - \exp\left(\frac{-2a^2}{R^2(z)}\right) \qquad \text{(S9)}$$

where $R(z)$ is given by:

$$R(z) = R_0\left[1 + \left(\frac{\lambda_{\mathrm{em}} z}{\pi R_0^2 n}\right)^2\right]^{1/2} \qquad \text{(S10)}$$

In the above equations *x, y, z* are Cartesian coordinates with *z* oriented along the optical axis, $\lambda_{\mathrm{ex}}$ referring to the excitation wavelength, $\lambda_{\mathrm{em}}$ referring to the center emission wavelength, *n* being the refractive index of the immersion medium (water), *a* denoting the radius of the confocal pinhole divided by magnification, and $w_0$ and $R_0$ being two initially unknown model parameters that are determined by the fit function. Taking together the parameterisation of the MDF for each focus and the solution of the diffusion equation (Green function), the following function is used to fit the ACFs and CCFs:

$$g(\tau,\delta) = g_\infty(\delta) + \frac{\varepsilon_1\varepsilon_2 c}{4}\left(\frac{\pi}{D\tau}\right)^{1/2} \int_{-\infty}^{+\infty} dz_1 \int_{-\infty}^{+\infty} dz_2 \frac{\kappa(z_1)\kappa(z_2)}{8D\tau + w^2(z_1) + w^2(z_2)} \cdot \exp\left[\frac{-(z_2-z_1)^2}{4D\tau} - \frac{2\delta^2}{8D\tau + w^2(z_1) + w^2(z_2)}\right] \tag{S11}$$

where $\tau$ is the lag time, $\delta$ the interfocal distance, $D$ the translational diffusion coefficient, $c$ the concentration of particles and $\varepsilon_1$ and $\varepsilon_2$ denote the overall detection efficiencies from both focal volumes. This model curve can be applied to the measured ACFs ($\delta$ = 0, $\varepsilon_1\varepsilon_2$ replaced by either $\varepsilon_1^2$ or $\varepsilon_2^2$) and CCFs simultaneously in a global fit with a linear least-square fitting approach including, among others, $\delta$ and $D$ as fit parameters. Computation and fitting is performed by a custom-made MATLAB routine. The interfocal distance $\delta$ can be determined by measuring dye solutions with known diffusion coefficients[6]. In this work, an interfocal distance of $\delta$ = 259 nm is used. Typical ACFs and CCFs curves of AF488-C(0)Aβ42 in 0.06 M GdnHCl can be fitted by a one-component fit model (Fig. S2).

**Photon Counting Histogram (PCH) analysis**

In the following we will only briefly describe the basics of PCH analysis. For more details we refer the reader to the paper by Chen *et al.*[7]. The number of photons of a light source with a constant intensity which are detected with a single-photon counting detector for a given bin time follows a Poissonian distribution. Any process that leads to fluctuations in the light intensity leads to a super-Poisson or sub-Poisson distribution of photon counts. Diffusion is the most important process which leads to these light fluctuations. Photon counting histogram analysis takes into account the fact that fluorescent molecules diffuse freely and independently from each other in a reference volume $V_0$. The probability to obtain $k$ photon counts from one molecule of brightness $\varepsilon$ which diffuses in a reference volume $V_0$ is obtained by summing all the Poissonians for the different positions $r$, weighted by the probability $p(r)$ that the fluorescent molecule occupies that position:

$$\begin{aligned} p^{(1)}(k,V_0,\varepsilon) &= \int_{V_0} \mathrm{Poi}(k,\varepsilon\,\mathrm{PSF}(r))\,p(r)\,dr \\ &= \frac{1}{V_0}\int_{V_0} \mathrm{Poi}(k,\varepsilon\,\mathrm{PSF}(r))\,dr \end{aligned} \tag{S12}$$

This equation can be generalized to the case of $N$ molecules:

$$p^{(N)}(k,V_0,\varepsilon)=\int\limits_{V_0}...\int\limits_{V_0} dr_1...dr_{\mathrm{N}}\, p(r_1)...p(r_{\mathrm{N}})\,\mathrm{Poi}\left(k,\varepsilon\sum_{i=1}^{N}\mathrm{PSF}(r_i)\right) \qquad \text{(S13)}$$

In case there is no particle in $V_0$ ($N$ = 0), the probability to receive $k$ photon counts $p^{(0)}(k,V_0,\varepsilon)$ is equal to zero except for $k$ = 0 where $p^{(0)}(k,V_0,\varepsilon)$ is equal to one, *i.e.*:

$$p^{(0)}(k,V_0,\varepsilon)=\begin{Bmatrix} 1, & k=0 \\ 0, & k>0 \end{Bmatrix} \qquad \text{(S14)}$$

In a real system, we deal with an open volume and thus with a fluctuating number of particles in $V_0$. The particle fluctuations in $V_0$ are assumed to obey Poissonian statistics. The final probability to obtain $k$ photon counts, given that $N$ particles are present in $V_0$, is consequently described by a weighted average of $p^{(N)}(k,V_0,\varepsilon)$ with this occupation number probability:

$$\Pi(k,\overline{N},\varepsilon)=\sum_{N=0}^{\infty} p^{(N)}(k,V_0,\varepsilon)\,\mathrm{Poi}(N,\overline{N}) \qquad \text{(S15)}$$

Therein, $\overline{N}$ denotes the average number of particles in the volume $V_0$. Here, we follow the convention proposed by Chen *et al.* to reference equation (S15) to the volume of the point spread function (equation (S2)). Consequently $\Pi(k,\overline{N},\varepsilon)$ becomes $\Pi(k,N_{\mathrm{PSF}},\varepsilon)$. In this case $N$ values from FCS and PCH analysis should coincide.

Out-of-focus emission can violate the 3D-Gaussian approximation of the observation volume profile as described by Perroud *et al.*[8]. Therefore, we applied a first-order correction as described in their paper:

$$p^{(1)}(k,V_0,\varepsilon)=\begin{cases} \dfrac{1}{(1+F)^2}\, p^{(1)}(k,V_0,\varepsilon)+\dfrac{\varepsilon F}{2\sqrt{2}}, & k=1 \\ \dfrac{1}{(1+F)^2}\, p^{(1)}(k,V_0,\varepsilon), & k>1 \end{cases} \qquad \text{(S16)}$$

where $F$ denotes the correction factor accounting for out-of-focus emission.

In case of the presence of two different particle species, the PCH is simply given as the convolution of the corresponding single species PCHs:

$$\Pi\,(k,N_{\mathrm{PSF},1},N_{\mathrm{PSF},2},\varepsilon_1,\varepsilon_2)=\Pi\,(k,N_{\mathrm{PSF},1},\varepsilon_1)\otimes\Pi\,(k,N_{\mathrm{PSF},2},\varepsilon_2) \qquad \text{(S17)}$$

Finally, a theoretical PCH model $\Pi(k, N_{\mathrm{PSF}}, \varepsilon)$ is fitted to the experimental photon counting histogram $\Pi_{\mathrm{exp}}$ using the reduced chi-squared criterium:

$$\chi_r^2 = \frac{\sum_{k=k_{\min}}^{k_{\max}} \left( M \frac{\Pi_{\mathrm{exp}} - \Pi(k, N_{\mathrm{PSF}}, \varepsilon)}{\sigma} \right)^2}{k_{\max} - k_{\min} - f_{\mathrm{p}}} \qquad \text{(S18)}$$

Herein, $k_{\min}$ and $k_{\max}$ denote the minimum and maximum count value in the experimental PCH, respectively. $f_{\mathrm{p}}$ stands for the number of fitting parameters. The number of total counts is given by *M*. $\sigma$ denotes the standard deviation. The fitting of the experimental PCHs was done using custom software written in MATLAB (this software will be made available on www.fluorfluc.com).

**Change in solvent-accessible surface area upon unfolding of AF488-C(0)Aβ42**

Knowing the cooperativity parameter *m* (equations (2), (3) and (4) in the manuscript), one can estimate the change in the solvent-accessible surface area (ΔSASA). Therefore, we use an empirical relationship reported by Myers *et al.*[9].

$$m = (958 \pm 270) + (0.23 \pm 0.02)\Delta\mathrm{SASA} \qquad \text{(S19)}$$

Herein the slope and intercept are in cal/(mol·M·Å$^2$) and cal/(mol·M), respectively. We reanalysed their data in order to get the parameters with their corresponding errors (equation (S19)). We found a slight deviation in the intercept, namely 958 in contrast to their value of 953. But due to the large error in the slope, this deviation is of negligible importance for the final results. The solvent-accessible surface area of an atom is typically defined in terms of the van der Waals radius of the atom and the radius of the solvent molecule[10].

A theoretical prediction of ΔSASA can be made on the basis of the number of residues in the peptide[9].

$$\Delta\mathrm{SASA} = -(907 \pm 248) + (93 \pm 1.5) \cdot (\#\text{of residues}) \qquad \text{(S20)}$$

Again, we reanalysed the data from Myers *et al.*[9] in order to obtain appropriate errors on the fit parameters (equation (S20)). The solvent-accessible surface area (SASA) of the native structural conformation of a protein can also be obtained from its crystal structure[10]. This of the denatured structural conformation is usually modelled by an extended polypeptide chain[9]. Calculating the change in solvent-accessible surface area (ΔSASA) using the *m*-value

according to equation (S19) returns ΔSASA = 5835 ± 5594 Å$^2$. The theoretical prediction of ΔSASA for a peptide of chain length 43 is 3092 ± 250 Å$^2$ (equation (S20)). However, the *m*- and ΔSASA-values seem to be in a reasonable range compared to other peptides[9,11] but interpretations should be done with caution due to the large errors in the estimation of ΔSASA. Nonetheless, the ΔSASA could be used as a benchmark value for other studies, especially for Aβ42 folding/unfolding molecular dynamics (MD) simulations.

**Robustness of 1fFCS towards increasing GdnHCl concentrations**

We explored that the axial and lateral laser beam waists were enlarged with increasing GdnHCl concentration (Fig. S3) and, as a result, lead to an increasing effective observation volume and to a decreasing correlation amplitude (Fig. S5). This is a consequence of the increasing refractive index of the corresponding GdnHCl solutions[5,12]. Thus, it is important to check that 1fFCS measurements are not affected by optical aberrations or by conformational changes of the dye AF488. Therefore, we applied 2fFCS, since this method is known to be robust towards refractive-index mismatches[13]. In the range of GdnHCl concentrations used in this work, the hydrodynamic radius of AF488 remains almost constant at about 0.9 nm (Fig. S6). This result shows that the unfolding of AF488 labelled C(0)Aβ42 with increasing GdnHCl concentration is not influenced by possible size changes of the dye since this occurs in the range between 1 nm to 1.3 nm. This also implies the validity to use AF488 maleimide as a calibrant in 1fFCS.

We measured a whole unfolding series of AF488-C(0)Aβ42 using 2fFCS and compared the corresponding unfolding curve with one obtained by 1fFCS in order to validate that the 1fFCS results are not biased by refractive-index changes (Fig. 3 A and B). Both unfolding curves show a similar course with a transition region between 1 and 2.5 M GdnHCl. The parameters $\Delta G^{H_2O}$ and *m* obtained by fitting these two unfolding curves to the two-state unfolding model (equation (1) in the manuscript) are very close together (Table 2).

The comparability of the 1fFCS and 2fFCS results shows that 1fFCS is sufficiently robust to track the unfolding of the small AF488-C(0)Aβ42. It is interesting to note that the error bars in Fig. 3 A and B tend to increase with increasing GdnHCl concentrations within one measurement series. The reasons for this may be manifold. One reason is that, besides the structural conversion of the peptide, the increasing viscosity leads to a decreasing diffusion coefficient *per se*. The contribution of each effect to the shift of the correlation curves changes with increasing GdnHCl concentration. The shift due to viscosity overwhelms the shift due to structural conversion at high GdnHCl concentrations (Fig. S7). Furthermore, an increasing GdnHCl concentration is accompanied with increasing optical aberrations, resulting in increasing laser beam parameters (Figs S3 and S4) and correspondingly to an increased observation volume and a decreasing correlation curve amplitude (Fig. S5). This,

in turn, leads to a higher intensity and to less distinct intensity fluctuations since more particles are observed.

Additionally, excitation and emission spectra may change upon changing the chemical environment of a fluorophore[14]. The excitation and emission spectra of AF488-C(0)Aβ42 conjugated AF488 and free AF488 are similar. Thus, it can be assumed that the conjugated fluorophore is in a similar environment as the free fluorophore and that it is not buried within a hydrophobic core or similar. A bathochromic shift in the excitation and emission spectra was observed when going from buffer (outer left spectrum for excitation and emission, Fig. S8 A) to highly concentrated GdnHCl solutions. Interestingly, similar shifts were also observed for the free dye (Fig. S8 B). The shift is more pronounced in the case of the free dye. This might indicate that the dye coupled to the peptide is slightly more shielded against the GdnHCl ions.

A bathochromic shift in the excitation and emission spectra (Fig. S8) results in a decrease in the detected intensity since the excitation and emission filter set was chosen according to the non-shifted spectrum at zero molar GdnHCl. As a consequence, the brightness decreased with increasing GdnHCl concentrations, as well as the signal-to-noise ratio, in the FCS measurements (see Table 1). This, to some extent, contributes to the larger error bars at higher GdnHCl concentrations (see Fig. 3 A and B).

## Supporting References


**1.** Finder, V.H., Vodopivec, I., Nitsch, R.M. & Glockshuber, R. The recombinant amyloid-beta peptide Abeta1-42 aggregates faster and is more neurotoxic than synthetic Abeta1-42. *J. Mol. Biol.* **396,** 9–18 (2010).

**2.** O'Connor, D.V. & Phillips, D. *Time-Correlated Single Photon Counting* (Academic Press Inc., London, 1984).

**3.** Wahl, M., Gregor, I., Patting, M. & Enderlein, J. Fast calculation of fluorescence correlation data with asynchronous time-correlated single-photon counting. *Opt. Express* **11,** 3583–3591 (2003).

**4.** Enderlein, J. & Gregor, I. Using fluorescence lifetime for discriminating detector afterpulsing in fluorescence-correlation spectroscopy. *Rev. Sci. Instrum.* **76,** 033102 (2005).

**5.** Dertinger, T. *et al.* Two-focus fluorescence correlation spectroscopy: a new tool for accurate and absolute diffusion measurements. *Chemphyschem* **8,** 433–443 (2007).

**6.** Müller, C.B. *et al.* Precise measurement of diffusion by multi-color dual-focus fluorescence correlation spectroscopy. *Europhys. Lett.* **83,** 46001 (2008).

**7.** Chen, Y., Müller, J.D., So, P.T. & Gratton, E. The photon counting histogram in fluorescence fluctuation spectroscopy. *Biophys. J.* **77,** 553–567 (1999).

**8.** Perroud, T.D., Huang, B., Wallace, M.I. & Zare, R.N. Photon counting histogram for one-photon excitation. *Chemphyschem* **4,** 1121–1123 (2003).

**9.** Myers, J.K., Pace, C.N. & Scholtz, J.M. Denaturant m values and heat capacity changes: relation to changes in accessible surface areas of protein unfolding, *Protein Sci.* **4,** 2138–2148 (1995).

**10.** Lee, B. & Richards, F.M. The interpretation of protein structures: estimation of static accessibility. *J. Mol. Biol.* **55,** 379–400 (1971).

**11.** Ni, C.-L., Shi, H.-P., Yu, H.-M., Chang, Y.-C. & Chen, Y.-R. Folding stability of amyloid-beta 40 monomer is an important determinant of the nucleation kinetics in fibrillization. *FASEB J.* **25,** 1390–1401 (2011).

**12.** Enderlein, J., Gregor, I., Patra, D., Dertinger, T. & Kaupp, U.B. Performance of fluorescence correlation spectroscopy for measuring diffusion and concentration. *Chemphyschem* **6,** 2324–2336 (2005).

**13.** Dertinger, T. *Two-focus fluorescence correlation spectroscopy*. Ph.D. thesis, Universität zu Köln, Germany (2007).

**14.** Lakowicz, J.R. *Principles of fluorescence spectroscopy* (3rd ed, Springer, New York, NY, 2010).

## Supporting Figures

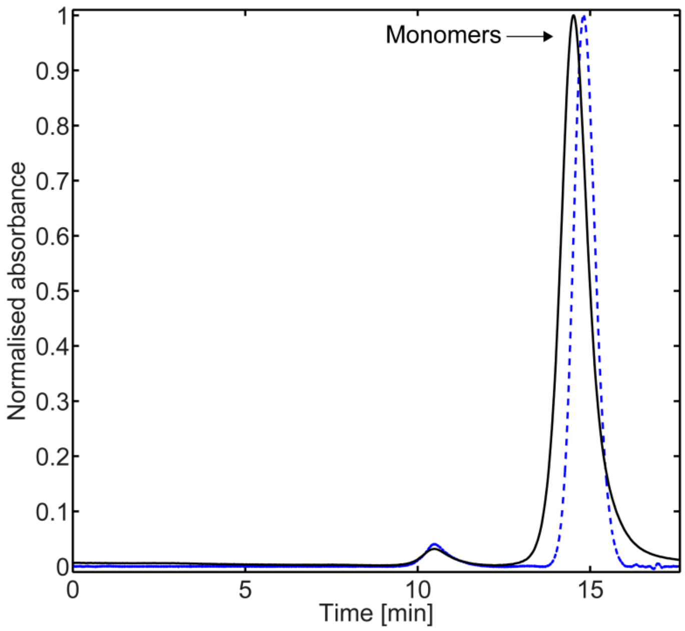


**Figure S1:** SEC monomer preparation of unmodified Aβ42 (blue dashed line) and AF488-C(0)Aβ42 (solid black line) on a Superdex 75 3.2/300 column (GE Healthcare). A small fraction of larger particles is eluted between 10 and 11 min. The bigger AF488-C(0)Aβ42 elutes earlier than the unmodified Aβ42.

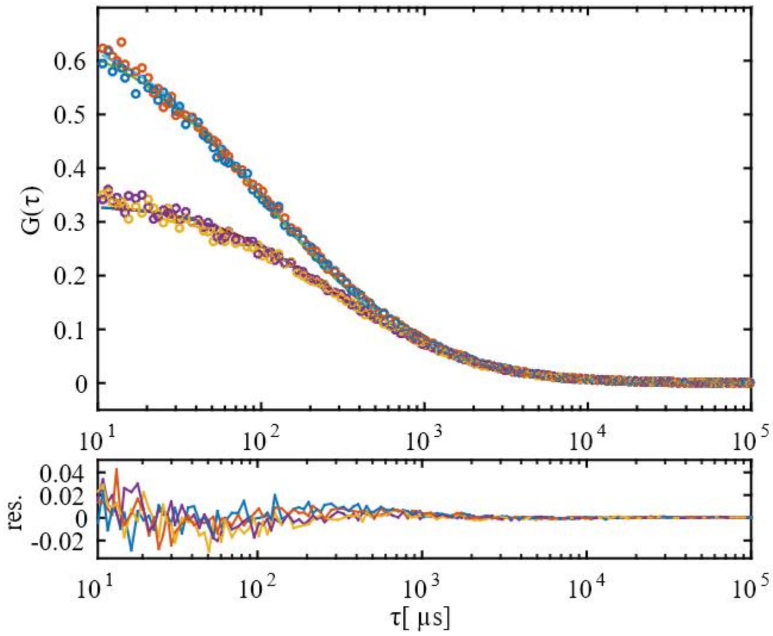


**Figure S2:** Typical ACF (blue and red ○) and CCF (orange and violet ○) curves as obtained by 2fFCS measurements of AF488-C(0)Aβ42 in 0.06 M GdnHCl. Solid lines denote best fit lines according to equation (S11). The corresponding residuals are shown at the bottom of this figure.

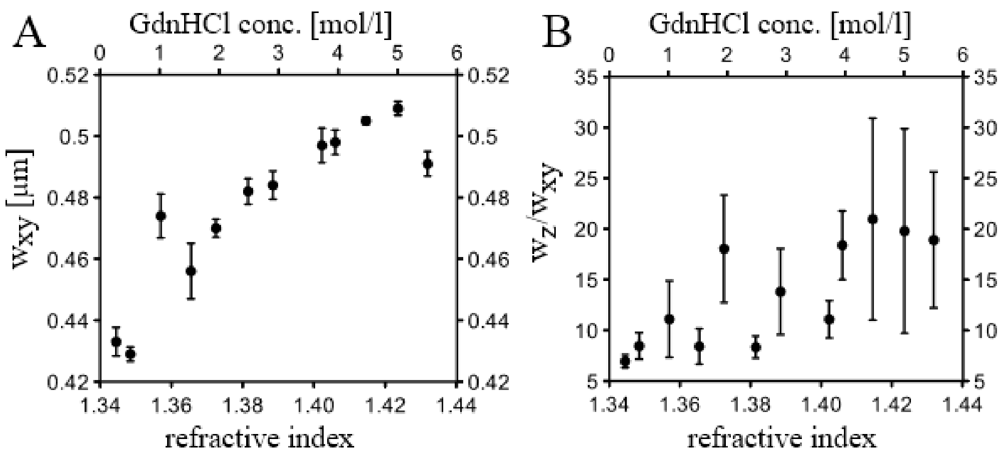


**Figure S3:** Effect of refractive index changes on laser beam parameters for 1fFCS. Due to the refractive index mismatch the laser beam became particularly elongated in the axial direction (*z*-direction) (B). Furthermore, a continuous increase of the lateral beam waist is also observed (A).

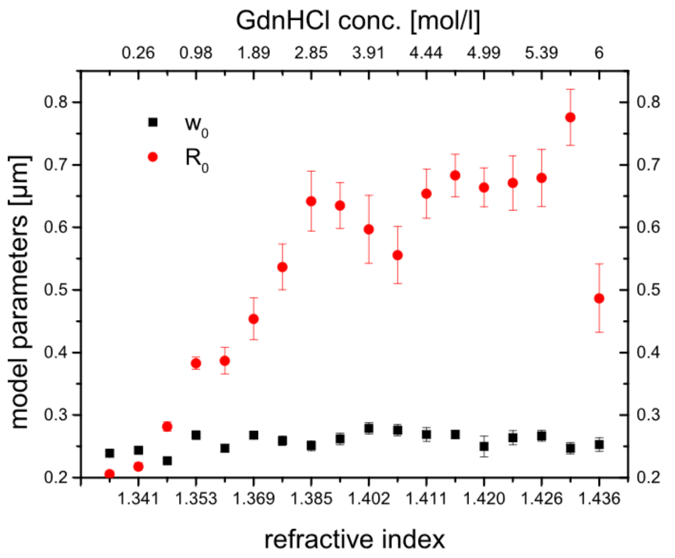


**Figure S4:** Effect of refractive index changes on model parameters $R_0$ and $w_0$ in 2fFCS.

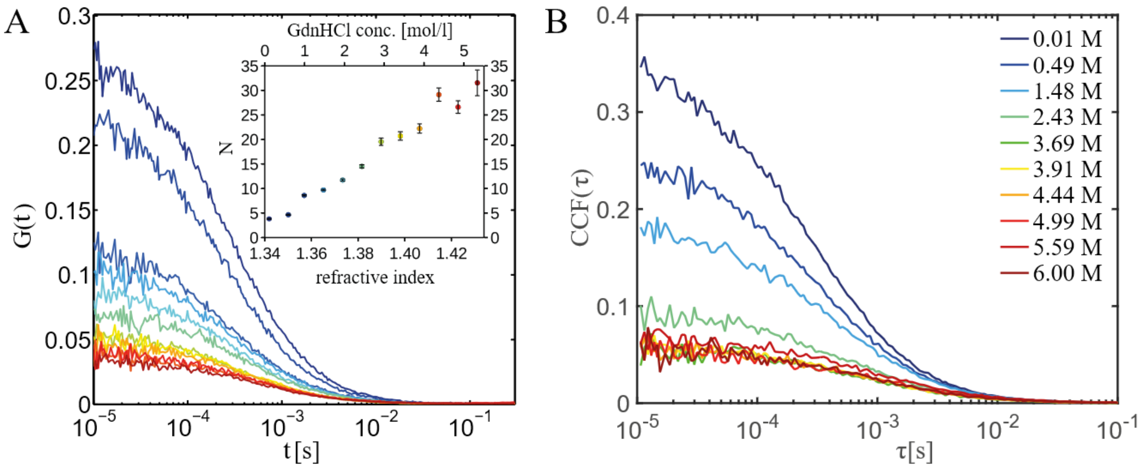


**Figure S5:** Selected correlation curves for 1fFCS (A) and 2fFCS (B) measurements of AF488-C(0)Aβ42 in solutions of variable GdnHCl concentrations. The increasing laser beam parameters entail increasing observation volumes and thus an increasing number of particles in these volumes (inset of A). The higher number of particles in turn leads to decreasing correlation amplitudes (A and B).

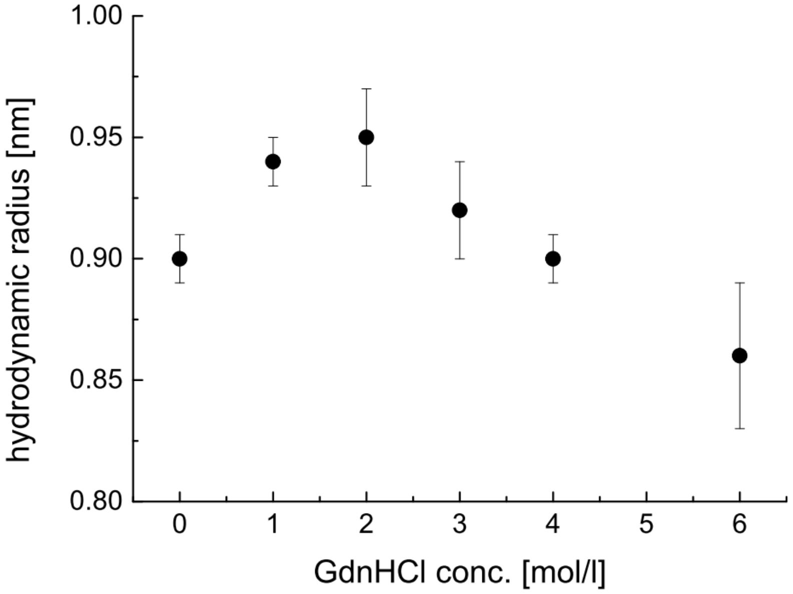


**Figure S6:** Hydrodynamic radii of AF488 maleimide in solutions of variable GdnHCl concentrations obtained by 2fFCS measurements.

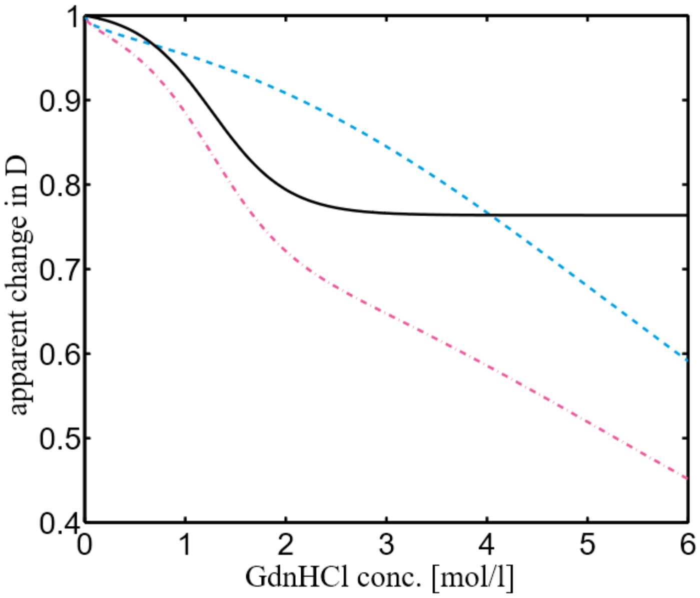


**Figure S7:** Relative change of the diffusion coefficient due to changes in viscosity (blue dashed line) and structural changes of the peptide (black solid line) only. The pink dash-dotted line represents the relative change of the diffusion coefficient due to the concurrence of both effects.

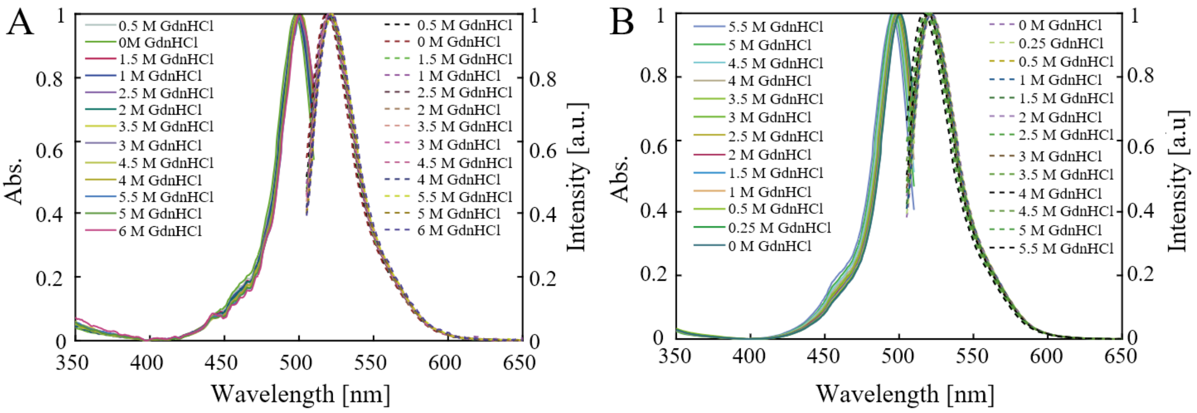


**Figure S8:** Excitation (solid lines) and emission spectra (dashed lines) of AF488-C(0)Aβ42 (A) and of AF488 maleimide in GdnHCl containing solutions (B). Figures were generated with ae software (available at www.fluortools.com).
